# Low serum mannose-binding lectin are associated with inflammation, new-onset diabetes mellitus and subclinical rejection after renal transplantation

**DOI:** 10.1186/1479-5876-9-S2-P30

**Published:** 2011-11-23

**Authors:** Francesc Moreso, Meritxell Ibernon, José M Fernández-Real, Wifredo Ricart, Daniel Seron

**Affiliations:** 1 Nephrology Dept., Hospital Universitari Vall d’Hebron, Barcelona, Spain; 2 Nephrology Dept., Hospital Germans Trias i Pujol, Badalona, Spain; 3 Dept. of Diabetes, Endocrinology and Nutrition, Institut d’Investigació Biomèdica de Girona, Girona, Spain

## Background

Infections, new onset diabetes mellitus (NODAT) and rejection are frequent complications after renal transplantation and may be related to innate immunity alterations. We evaluate the relationship between serum mannose-binding lectin (MBL) levels, chronic inflammation, infection, NODAT and subclinical rejection after renal transplantation.

## Patients and methods

Between March 2005 and October 2006 consecutive non-diabetic renal transplant recipients were recruited. Serum levels of MBL, soluble tumor necrosis factor receptor 2 (sTNFR2) and neutrophil gelatinase associated lipocalin (NGAL) were determined before transplant and at 1 and 3 months. An oral glucose tolerance test was performed at 3 months. A surveillance 3-month renal biopsy was performed in a subset of 60 patients with stable renal function.

## Results

A total of 125 patients were recruited and 111 had a functioning graft at 3 months. MBL serum levels remained unchanged following transplantation. Subjects with low MBL (lower tertile) had higher pretransplant sTNFR2 (40 ± 13 vs. 35 ± 11 ng/ml, p=0.05) and NGAL (638 ± 114 vs. 553 ± 185 ng/ml, p=0.03), an increased incidence of bacterial/fungal infection (p=0.021) and an increased prevalence of NODAT at 3 months (44.4 vs 22.6%, p=0.01). Multivariate analysis confirmed that MBL was a risk factor for NODAT (relative risk: 3.04, 95% confidence interval: 1.18-7.81; p=0.021) adjusting for age, pre-transplant impaired fasting glucose and body mass index. Subclinical rejection in the 3-month surveillance biopsy was observed in 7 of 18 (38.9%) low MBL patients and in 3 of 42 (7.1%) high MBL patients (p=0.005). Induction and maintenance immunosuppression was not different in patients with low and high MBL levels.

## Conclusion

Low MBL serum levels in renal transplants are associated with major outcome variable after renal transplantation such as bacterial/fungal infections, NODAT and subclinical rejection. These results suggest that alterations of the innate immunity may play an important role in renal transplantation.

